# Focused ultrasound as a novel strategy for noninvasive gene delivery to retinal Müller glia

**DOI:** 10.7150/thno.42611

**Published:** 2020-02-10

**Authors:** Yacine Touahri, Rajiv Dixit, Rikke Hahn Kofoed, Kristina Mikloska, EunJee Park, Reza Raeisossadati, Kelly Markham-Coultes, Luke Ajay David, Hibo Rijal, Jiayi Zhao, Madelaine Lynch, Kullervo Hynynen, Isabelle Aubert, Carol Schuurmans

**Affiliations:** 1 Biological Sciences Platform, Sunnybrook Research Institute, Toronto, Ontario, Canada.; 2 Department of Biochemistry, University of Toronto, Toronto, Ontario, Canada.; 3 Physical Sciences Platform, Sunnybrook Research Institute, Toronto, Ontario, Canada.; 4 Department of Laboratory Medicine and Pathobiology, University of Toronto, Toronto, Ontario, Canada; 5 Department of Medical Biophysics, University of Toronto, Toronto, Ontario, Canada.

**Keywords:** retina, AAV-PHP.eB, AAV2/8, GFAP promoter, Müller glia, gene therapy, blood-retinal-barrier

## Abstract

Müller glia are specialized retinal cells with stem cell properties in fish and frogs but not in mammals. Current efforts to develop gene therapies to activate mammalian Müller glia for retinal repair will require safe and effective delivery strategies for recombinant adeno-associated viruses (AAVs), vectors of choice for clinical translation. Intravitreal and subretinal injections are currently used for AAV gene delivery in the eye, but less invasive methods efficiently targeting Müller glia have yet to be developed.

**Methods**: As gene delivery strategies have been more extensively studied in the brain, to validate our vectors, we initially compared the glial tropism of AAV-PHP.eB, an AAV9 that crosses the blood-brain and blood-retinal barriers, for its ability to drive fluorescent protein expression in glial cells in both the brain and retina. We then tested the glial transduction of AAV2/8-GFAP-mCherry, a virus that does not cross blood-brain and blood-retinal barriers, for its effectiveness in transducing Müller glia in murine retinal explants *ex vivo*. For *in vivo* assays we used larger rat eyes, performing invasive intravitreal injections, and non-invasive intravenous delivery using focused ultrasound (FUS) (pressure amplitude: 0.360 - 0.84 MPa) and microbubbles (Definity, 0.2 ml/kg).

**Results**: We showed that AAV-PHP.eB carrying a ubiquitous promoter (CAG) and green fluorescent protein (GFP) reporter, readily crossed the blood-brain and blood-retinal barriers after intravenous delivery in mice. However, murine Müller glia did not express GFP, suggesting that they were not transduced by AAV-PHP.eB. We thus tested an AAV2/8 variant, which was selected based on its safety record in multiple clinical trials, adding a glial fibrillary acidic protein (GFAP) promoter and mCherry (red fluorescent protein) reporter. We confirmed the glial specificity of AAV2/8-GFAP-mCherry, showing effective expression of mCherry in astrocytes after intracranial injection in the mouse brain, and of Müller glia in murine retinal explants. For *in vivo* experiments we switched to rats because of their larger size, injecting AAV2/8-GFAP-mCherry intravitreally, an invasive procedure, demonstrating passage across the inner limiting membrane, leading to Müller glia transduction. We then tested an alternative non-invasive delivery approach targeting a different barrier - the inner blood-retinal-barrier, applying focused ultrasound (FUS) to the retina after intravenous injection of AAV2/8 and microbubbles in rats, using magnetic resonance imaging (MRI) for FUS targeting. FUS permeabilized the rat blood-retinal-barrier and allowed the passage of macromolecules to the retina (Evans blue, IgG, IgM), with minimal extravasation of platelets and red blood cells. Intravenous injection of microbubbles and AAV2/8-GFAP-mCherry followed by FUS resulted in mCherry expression in rat Müller glia. However, systemic delivery of AAV2/8 also had off-target effects, transducing several murine peripheral organs, particularly the liver.

**Conclusions**: Retinal permeabilisation via FUS in the presence of microbubbles is effective for delivering AAV2/8 across the inner blood-retinal-barrier, targeting Müller glia, which is less invasive than intravitreal injections that bypass the inner limiting membrane. However, implementing FUS in the clinic will require a comprehensive consideration of any off-target tropism of the AAV in peripheral organs, combined ideally, with the development of Müller glia-specific promoters.

## Introduction

Retinal degenerative diseases such as age-related macular degeneration (AMD) lead to photoreceptor cell death and irreversible vision loss 1. Replacing lost photoreceptors is essential to restore vision, but like other regions of the central nervous system (CNS), new neurons, including photoreceptors, are not made in adult mammalian eyes. In contrast, spontaneous, endogenous repair occurs in the retinas of cold-blooded vertebrates through the latent stem cell properties of Müller glia 2. Müller glia are 'activated' in response to injury in organisms such as teleost fish and frogs, leading to de-differentiation, re-entry into the cell cycle, and re-differentiation to replace lost retinal cells 3. An obstacle to the repair response by mammalian Müller glia is that they instead preferentially undergo reactive gliosis when injured, a neuroprotective response associated with cellular hypertrophy, barrier formation around the injury site, the secretion of neuronal pro-survival factors, antioxidants, and pro-endothelial molecules, and the increased expression of intermediate filament genes, such as glial fibrillary associated protein (GFAP) 3, 4. Recently, several studies reported successful Müller glia-based regeneration in mammals by overexpressing specific gene combinations *in vivo* 5-8. This significant advance has created a unique and promising opportunity for cell replacement by activating endogenous Müller glia, and propelled the need to develop a safe and efficient delivery strategy for gene therapies targeting these cells.

The main limitation to developing therapeutics for retinal diseases is that access to the retina for large macromolecules is limited by three natural barriers; the inner (ILM) and outer (OLM) limiting membranes and the blood-retinal barrier (BRB). The ILM is a basement membrane delimited by the basal endfeet of Müller glia that forms a barrier to therapeutics delivered intravitreally 9. The OLM is comprised of Müller glia endfeet attached to photoreceptor outer segments via adherens junctions and desmosomes, and is a barrier to subretinal delivery 10. The inner BRB is made up of tight junctions between astrocytes, pericytes and endothelial cells, and is a barrier to systemic delivery, preventing passage from the circulation into the retina through the deep, intermediate and superficial vascular plexi 9, 11. Finally, the outer BRB is the retinal pigment epithelium (RPE), an epithelial layer that separates the retina from choroidal blood vessels.

Recombinant adeno-associated-viruses (AAVs) are the vehicles of choice for gene therapy as they support high gene expression levels and are non-integrating, non-replicating, and show tropism for specific cell types 12. AAV gene therapies have begun to be applied to retinal diseases in clinical trials 13-16, including Lebers congenital amaurosis, for which viral delivery of a corrected RPE65 gene (Luxturna) has been approved 17. Gene therapy has also been established for choroideremia, targeting the macula 18. In both instances, subretinal AAV injections are required, which can have complications, including retinal detachment and macular thinning. In contrast, murine Müller glia are targeted via intravitreal injections, with successful transduction with several capsids, including AAV2/5 19, AAV2/6 20, 21, AAV-ShH10 (related to AAV6) 21-23 and AAV2/9 22. The other part of the equation is promoter specificity, which can either be ubiquitous (e.g. CAG) or cell type-specific. The most frequently used promoter to drive expression in Müller glia is the GFAP promoter, which is expressed in a subset of Müller glia that are reactive in response to injury (e.g., 24).

While intravitreal injections are successful, they are also invasive, with some studies reporting significant complications, including endophthalmitis, inflammation, hemorrhage, retinal detachment and RPE tears 25, 26. The ideal approach would be a non-invasive delivery strategy. Therefore, we investigated whether focused ultrasound (FUS), in combination with microbubbles injected intravenously, could serve as an alternative non-invasive approach to efficiently deliver genes, administered systemically, across the BRB and into Müller glia. Mechanistically, the acoustic energy generated by an ultrasound transducer is captured by circulating microbubbles, which oscillate, interact with blood vessel walls, and temporarily disrupt tight junctions 27, 28. In the brain, this type of FUS application has been performed transcranially to safely, locally, and briefly increase the permeability of the blood-brain barrier (BBB) 29, 30. In preclinical models, FUS-BBB modulation allows macromolecules - endogenous or administered - as well as progenitor cells and gene vectors injected intravenously, to pass from the blood through the BBB to enter FUS-targeted brain regions 31-35. One study also reported permeabilization of the rat BRB using FUS 36. However, to date, the delivery of therapeutics or AAVs with FUS across the BRB has not been reported.

We first tested whether systemic delivery of an AAV9 derivative (AAV-PHP.eB), known to cross the BBB and BRB 37, could target Müller glia, but despite widespread retinal transduction, retinal glia were not targeted. We thus switched to an AAV2/8 virus (inverted terminal repeat of type 2 and capsid of type 8) driving expression of an mCherry reporter (red fluorescent protein) 38 under the control of a GFAP promoter, which transduced Müller glia* in vitro* in mouse retinal explants*,* and *in vivo* in rat eyes following intravitreal injections. Finally, we demonstrated that FUS coupled with microubbles could permeabilize the rat BRB, allowing the transfer of blood-borne macromolecules and systemically injected AAV2/8-GFAP-mcherry to retinal tissue. Our study highlights: (1) the potential of AAV-PHP.eB to cross the BRB and transduce a subpopulation of inner retinal cells, including ganglion cells but not Müller glia; and (2) the efficacy of AAV2/8-hGFAP-mCherry in transducing Müller glia when injected intravitreally (to cross the inner limiting membrane) or intravenously, when coupled with FUS and microbubbles (to cross the inner BRB).

## Results

### AAV-PHP.eB capsid efficiently targets the brain and retina but not Müller glia

Most AAVs do not cross the BBB or BRB, requiring invasive intracranial or intravitreal/subretinal delivery strategies, respectively 39. However, systemic delivery of the novel AAV9 variant AAV-PHP.B by intravenous injection leads to widespread transduction of the brain and retina in C57BL/6 mice 37. The ability of AAV-PHP.B to transduce cells in the murine brain was further improved with the AAV-PHP.eB variant 40. While the AAV-PHP.B variant was demonstrated to transduce the retina 37, its cellular tropism was not investigated, and the ability of the novel AAV-PHP.eB capsid to target the retina was not determined 40. We thus tested whether AAV-PHP.eB could transduce retinal Müller glia, our cells of interest, which could allow a non-invasive delivery strategy for gene therapies.

**Figure 1:**
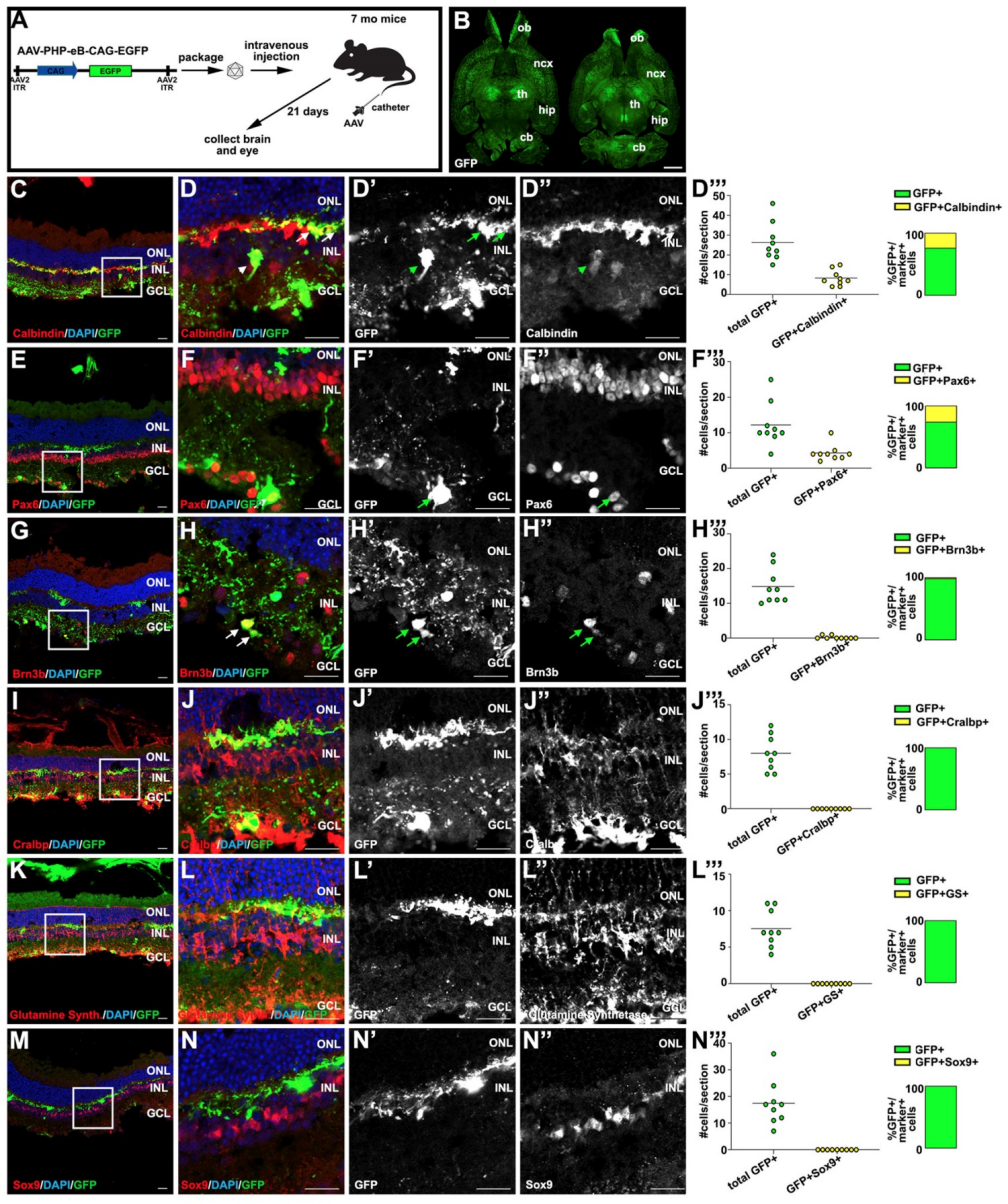
** Transduction profiling after systemic delivery of AAV-PHP.eB in the brain and retina.** (A) Schematic illustration of AAV-PHP.eB viral construct and experimental design, depicting intravenous injection through the tail vein of 7 months C3H/He-C57BL/6 mice. (B) GFP expression in transverse brain sections 21 days post-AAV-PHP.eB transduction. (C-N) Analysis of GFP expression in retinal sections 21 days post-AAV-PHP.eB transduction, examining co-expression with calbindin (horizontal and amacrine cell subtypes) (C,D-D''), Pax6 (amacrine and some retinal ganglion cells) (E,F-F''), Brn3b (retinal ganglion cells) (G,H-H''), Cralbp (Müller glia) (I,J-J''), Glutamine synthetase (Müller glia) (K,L-L'') and Sox9 (Müller glia) (M,N-N''). Quantification of GFP+ cells that express Calbindin+, Pax6+, Brn3b+, Glutamine Synthetase (GS)+ and Sox9+, respectively per section (D''', F''', H''', J''', L''', N'''). Arrows mark double positive cells. Blue is DAPI counterstain. Boxed areas in C,E,G,I,K,M are magnified in D-D'', F-F'', H-H'', J-J'', L-L'' and N-N'', respectively. cb, cerebellum; GCL, ganglion cell layer; hip, hippocampus; INL, inner nuclear layer; ncx, neocortex; ONL, outer nuclear layer; th, thalamus. (Scale bar in B=2000 μm; and in all other figures=25 μm).

To investigate the ability of AAV-PHP.eB to transduce different cell types in the brain and retina, we performed intravenous injections in adult C3H/He-C57BL/6 mice, a hybrid strain often used to model neurodegenerative disorders 41. As AAV-PHP.eB does not have the same widespread neural-tropism in all mouse strains 42, we first confirmed that the AAV-PHP.eB capsid could transduce cells in the brain in this mouse strain. 7 month old hybrid C3H/He-C57BL/6 female mice were injected intravenously with 3.33x10^9^ genome copies (GC)/g of AAV-PHP.eB-CAG-GFP and 3 weeks later, the brain and eyes were harvested for immunostaining (Fig. 1A). The CAG promoter/enhancer was used to drive GFP expression as this regulatory element drives gene expression in neuronal and glial cells in the CNS 43. As reported 40, we observed robust GFP expression in scattered cells throughout the brain, indicating that AAV-PHP.eB could cross the BBB, and supporting the idea that the CAG promoter was able to drive ubiquitous expression of the GFP transgene (Fig. 1B).

Having bypassed the BRB, AAV-PHP.eB transduced inner retinal cells, which expressed GFP (Fig. 1C-N'), similar to the observations made for AAV-PHP.B 37. The inner nuclear layer (INL) is populated not only by Müller glia, but also by amacrine, bipolar and horizontal cell interneurons, prompting us to use cell type-specific markers to identify (Fig. 1C-N') and quantify (Fig. 1D'''-N''') the inner retinal cells transduced by AAV-PHP.eB. GFP colocalized with calbindin^+^ horizontal cell processes in the outer plexiform layer (arrows; Fig. 1C,D-D''', 31.8% GFP+ cells), and in some calbindin^+^ amacrine cells in the INL (arrowheads). However, most Pax6^+^ amacrine cells in the INL were not GFP^+^ and were thus not targeted by AAV-PHP.eB (Fig. 1E,F-F'''). Instead, Pax6^+^GFP^+^ cells were detected in the ganglion cell layer (GCL) (Fig. 1E,F-F''', 35.4% GFP+ cells), which is populated by displaced amacrine cells and ganglion cells. We confirmed that AAV-PHP.eB targets ganglion cells by co-staining with Brn3b (Fig. 1G,H-H''', 1.4% GFP+ cells). In contrast, GFP did not co-localize with several Müller glia-specific markers, including Cralbp (Fig. 1I,J-J''', 0% GFP+ cells), glutamine synthetase (Fig. 1K,L-L''', 0% GFP+ cells) and Sox9 (Fig. 1M,N-N''', 0% GFP+ cells), which failed to co-localize in the cell bodies or fine processes that these cells normally extend across the retina.

Thus, while AAV-PHP.eB can transduce a number of retinal cell types after systemic delivery, primarily horizontal, some amacrine and ganglion cells, it is not efficient at transducing Müller glia, our target cell population for therapeutic purposes.

### Validating glial targeting by AAV2/8-GFAP-mCherry in the brain and retina

**Figure 2:**
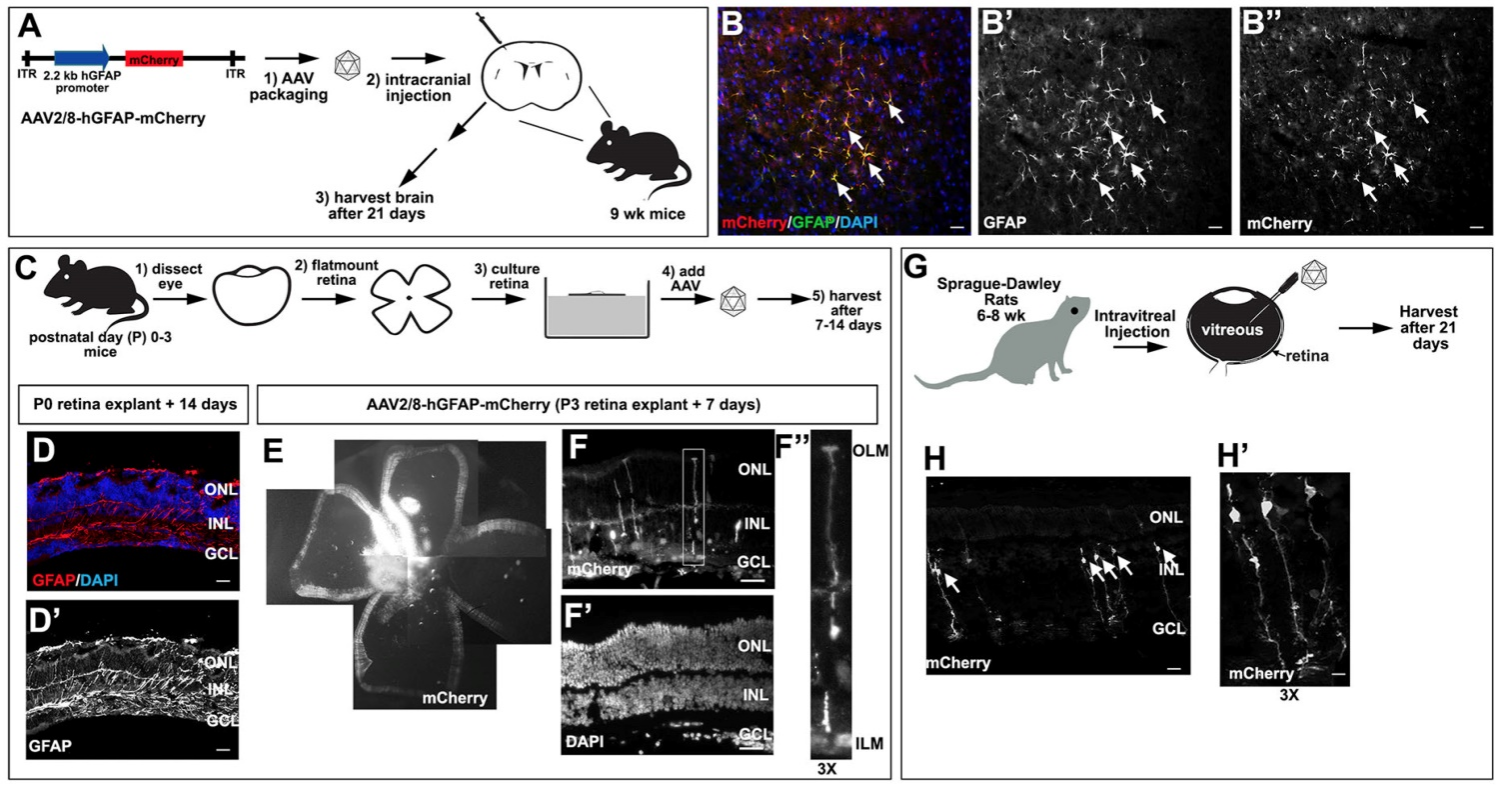
** Validation of AAV2/8-GFAP-mCherry transduction of glial cells in the brain and retina.** (A) Schematic of AAV2/8-GFAP-mCherry construct and experimental design, depicting intracranial injections in 9 week old mice. (B-B'') mCherry expression in GFAP expressing astrocytes in the neocortex of AAV2/8-GFAP-mCherry transduced mouse brains 3 weeks post-transduction. (C-F) Experimental design for the transduction of murine P0-P3 retinal explants with AAV2/8-GFAP-mCherry (C). Analysis of GFAP expression in P0 mouse retinal explants cultured 14 days *in vitro* (D,D'). Blue is DAPI counterstain. Analysis of the expression of mCherry (E,F,F'') and DAPI (F') in P3 retinal explants cultured for 7 days post-transduction. F'' is a high magnification image of boxed area in F. (G-H) Experimental design for the intravitreal injection of rat retinas with AAV2/8-GFAP-mCherry (G). Expression of mCherry in rat retinas 21 days post intravitreal injection of AAV2/8-GFAP-mCherry (H,H'). Arrows mark cells with typical Müller glia morphology. H' is a high magnification of H. GCL, ganglion cell layer; INL, inner nuclear layer; ONL, outer nuclear layer. (Scale bars =25 μm).

Given that AAV-PHP.eB-CAG-GFP did not transduce Müller glia when delivered systemically (Fig. 1), we set out to identify an AAV vector that could target these retinal glial cells, which would allow us to test other non-invasive delivery strategies. Two main factors are considered for cell-type specific expression using AAVs: the capsid confers cell tropism for transduction, and the promoter controls the specificity of transgene expression 40. We selected AAV2/8 as our vector of choice based on several criteria. First, AAV2/8, unlike other more recently engineered vectors with known tropism for Müller glia (e.g. Shh10 21, 44, 45), has already passed safety tests and according to ClinicalTrials.gov is in eight clinical trials for various diseases (NCT03533673, NCT03533673, etc.). Second, AAV2/8 has known tropism for other glial cell types, such as astrocytes in the brain 46. Third, AAV2/8 has a high transduction efficiency in the retina, meaning that less virus might be needed for clinical translation, and importantly, intravitreal injections of AAV2/8 do not impair retinal function 47. Finally, while a previous study suggested that AAV2/8 does not transduce retinal Müller glia efficiently 48, the authors used a CMV promoter that is not highly expressed in glial cells 43. Thus, the potential tropism of AAV2/8 for Müller glia was not tested under ideal conditions. We thus combined a 2.2 kb human (h) GFAP promoter sequence, as murine and human GFAP promoters drive GFP expression in retinal Müller glia 20, 49, 50, with an mCherry reporter in AAV2/8 (Fig. 2A).

We first confirmed the glial tropism of our selected construct by validating that AAV2/8-hGFAP-mCherry could transduce murine brain astrocytes. We thus performed intracranial injections of AAV2/8-hGFAP-mCherry into the motor cortex of 9 week old C57Bl/6 mice (n=3) and examined mCherry expression in brain sections after 21 days (Fig. 2A). mCherry expression was observed in a subset of GFAP^+^ astrocytes that had the typical stellate morphology of reactive glial cells (Fig. 2B-B''), confirming that AAV2/8-hGFAP-mCherry could efficiently transduce astrocytes in the mouse brain, as previously reported 46.

We then asked whether AAV2/8-hGFAP-mCherry was able to transduce Müller glia using a mouse retinal explant system (Fig. 2C). Importantly, this system is ideal to test AAV2/8-hGFAP-mCherry tropism and cell type-specific expression as there is an injury response and a strong GFAP upregulation when retinas are explanted and cultured *in vitro* 51. To confirm that Müller glia undergo reactive gliosis in explants, we examined GFAP expression in postnatal day (P) 0 mouse retinas cultured for 14 days *in vitro* as flatmounts, revealing a robust upregulation in expression of this intermediate filament (Fig. 2D,D'). We next tested AAV2/8-hGFAP-mCherry infectivity in P3 mouse retinal explants. Low dose AAV2/8-hGFAP-mCherry was pipetted onto the explants and 7 days later, explants were harvested and imaged for mCherry epifluorescence, revealing a high level of infectivity of this viral serotype in the retina (Fig. 2E). Sectioning of the transduced retinal explants revealed that mCherry expression was preferentially detected in cells with a typical Müller glia-like morphology, with their cell bodies in the INL, and projections to the ILM and OLM, where endfeet structures were anchored (Fig. 2F-F'').

Finally, we tested the ability of AAV2/8-hGFAP-mCherry to transduce Müller glia when injected intravitreally, the most common route of AAV delivery to the retina 48; and with FUS 36, as a new non-invasive approach for gene delivery to the retina. For these experiments, we utlized rats to increase technical feasibility and improve FUS reproducibility, due to the larger size of rat eyes, compared to mouse. We injected two Sprague-Dawley rats (6 weeks old) intravitreally with 2 μL of AAV2/8-GFAP-mCherry at 2×10^12^ GC/ml (Fig. 2G). 21 days later, eyes were collected and sectioned, and mCherry expression was examined (Fig. 2H,H'). We observed specific expression of mCherry in radially polarized cells, with cell bodies located in the INL and processes extending to the OLM and ILM (Fig. 2H,H'), a unique morphology characteristic of Müller glia 52, 53.

AAV2/8-hGFAP-mCherry, which incorporates hGFAP promoter and an AAV2/8 serotype, can thus infect and express mCherry in Müller glia, *ex-vivo* in mice and *in vivo* in rats*.*

### Developing focused ultrasound to permeabilize the blood-retinal-barrier

**Figure 3:**
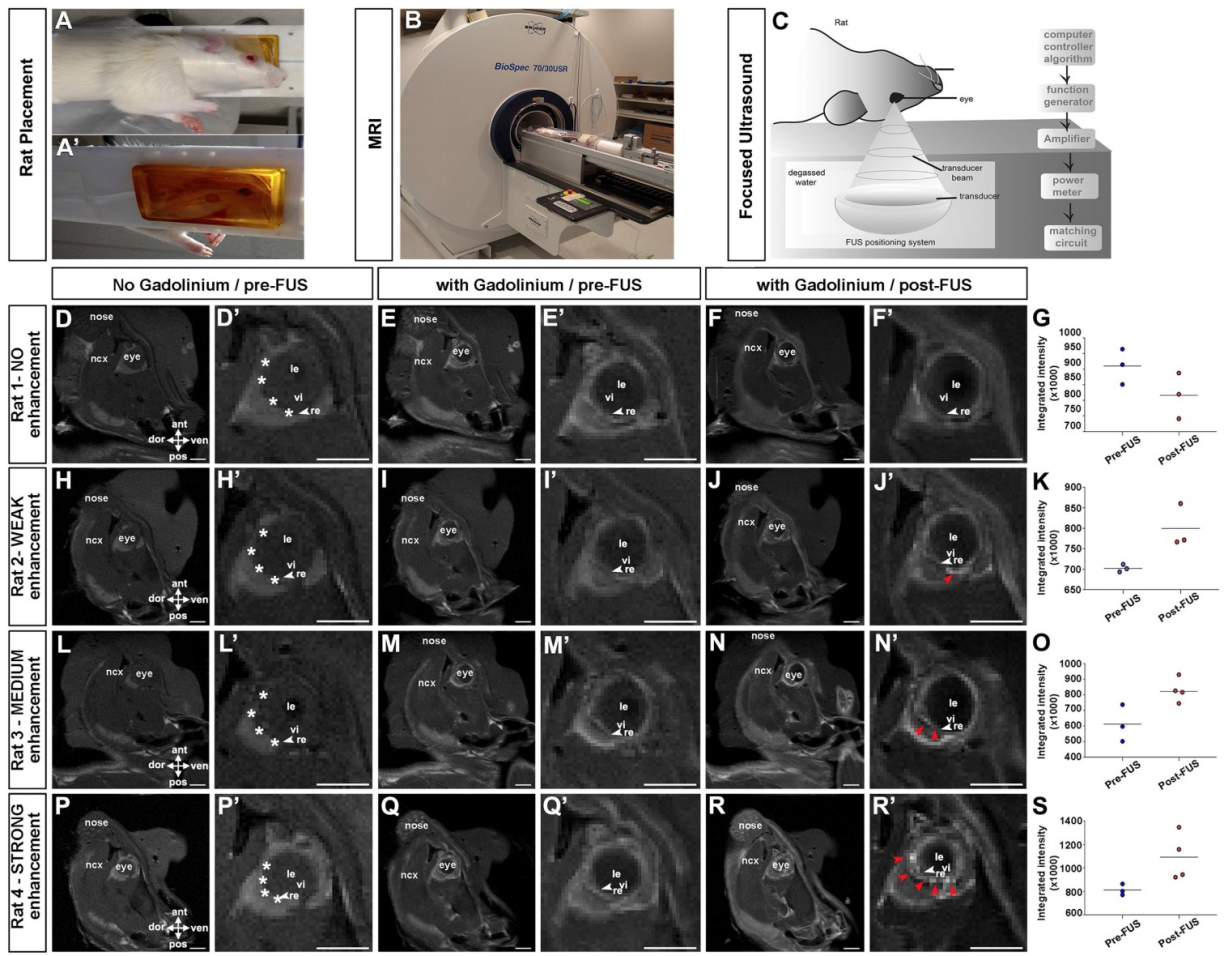
** MR imaging showing contrast enhancement in the retina after focused ultrasound.** (A-C) Animal setup for MRI guided FUS; rats were laid in a supine position on a sledge that is MRI and FUS compatible, with an angiocatheter inserted into the tail vein (A). Rats were introduced into the MRI scanner for imaging pre- and post FUS (B). Schematic illustration of FUS device (C). (D,D',H,H',L,L',P,P') T1-weighted MR images of rats taken before the administration of gadolinium contrast agent, 15 minutes post gadolinium exposure (E,E',I,I',M,M',Q,Q') and immediately after FUS (F,F',J,J',N,N',R,R'). Shown are 4/6 rats tested, showing no enhancement (E-F') weak (I-J'), medium (M-N') and strong (Q-R') enhancement. White asterisks in D',H',L'P' show approximate locations of four selected coordinates for FUS. Red arrowheads in J',N',R' show enhancement post FUS. Gadolinium enhancement quantification pre and post FUS (G,K,O,S). ant, anterior; dor, dorsal; le, lens; ncx, neocortex; pos, posterior; re, retina; ven, ventral; vi, vitreous. (Scale bars= 5 mm).

Our goal was to develop a non-invasive method to deliver gene therapies to Müller glia. Given our prior success permeabilizing the BBB with FUS 54, 55, and a documented study demonstrating that FUS could also be used to transiently permeabilize the BRB 36, we set out to establish FUS parameters for BRB opening that would allow viral delivery. We used rats, based on the size of their eyes to facilitate the procedure 36. A magnetic resonance (MR) imaging-compatible FUS system (operationally similar to that used in 56), was used to permeabilize the BRB in six animals. Rats were anesthetized and placed on their side (one eye down) (Fig. 3A-A') on a removable sled, which was positioned in a 7 Tesla MR machine (Fig. 3B). Baseline T1-weighted MR images were captured before (Fig. 3D,D',H,H',L,L',P,P') and after (Fig. 3E,E',I,I',M,M',Q,Q') the administration of gadolinium contrast agent. MR images were used to select four ultrasound targets on the temporal side of the retina in the right eye (asterisks in Fig. 3D',H',L',P), with the left eye serving as a control.

Once FUS targets were identified, the sled was moved outside of the MR scanner and placed on a custom FUS machine for sonication, as previously described 57. To permeabilize the BRB, microbubbles were injected intravenously through a tail vein catheter and the eye was exposed to a focused transducer, used at a frequency of 1.1 MHz with a feedback controller 58 (Fig. 3C). Rat eyes have an approximate diameter of 6-7 mm. The transducer focused the ultrasound beam to a spot of 0.8 mm diameter and 5 mm length (80% pressure contour), at a distance of ~60 mm from the corneal surface, allowing precise targeting using the MR images. The effect of the ultrasound on the microbubbles was verified by monitoring the acoustic emission from the exposed microbubbles using a hydrophone located in the middle of the transducer.

The pressure was gradually increased in each burst until sub-harmonic emissions were detected and then continued at 50% of the scaling level. This method has previously been shown to induce a post-sonication enhancement in gadolinium of 19% in rat brains 58. Clear gadolinium enhancement was observed (Fig. 3F',J',N',R') and quantified (Fig. 3G,K,O,S) by comparing contrast-enhanced T1-weighted MR images with and without gadolinium in three out of six rats (red arrowheads in Fig. 3J',N',R'), indicative of BRB permeabilization. Based on the MR images we classified gadolinium enhancement as not present (3/6) (Fig. 3F,F'), weak (1/6) (Fig. 3J,J') medium (1/6) (Fig. 3N,N') and strong (1/6) (Fig. 3R,R') (see Table S1 for summary).

### Using FUS to deliver systemic macromolecules into the retina

**Figure 4:**
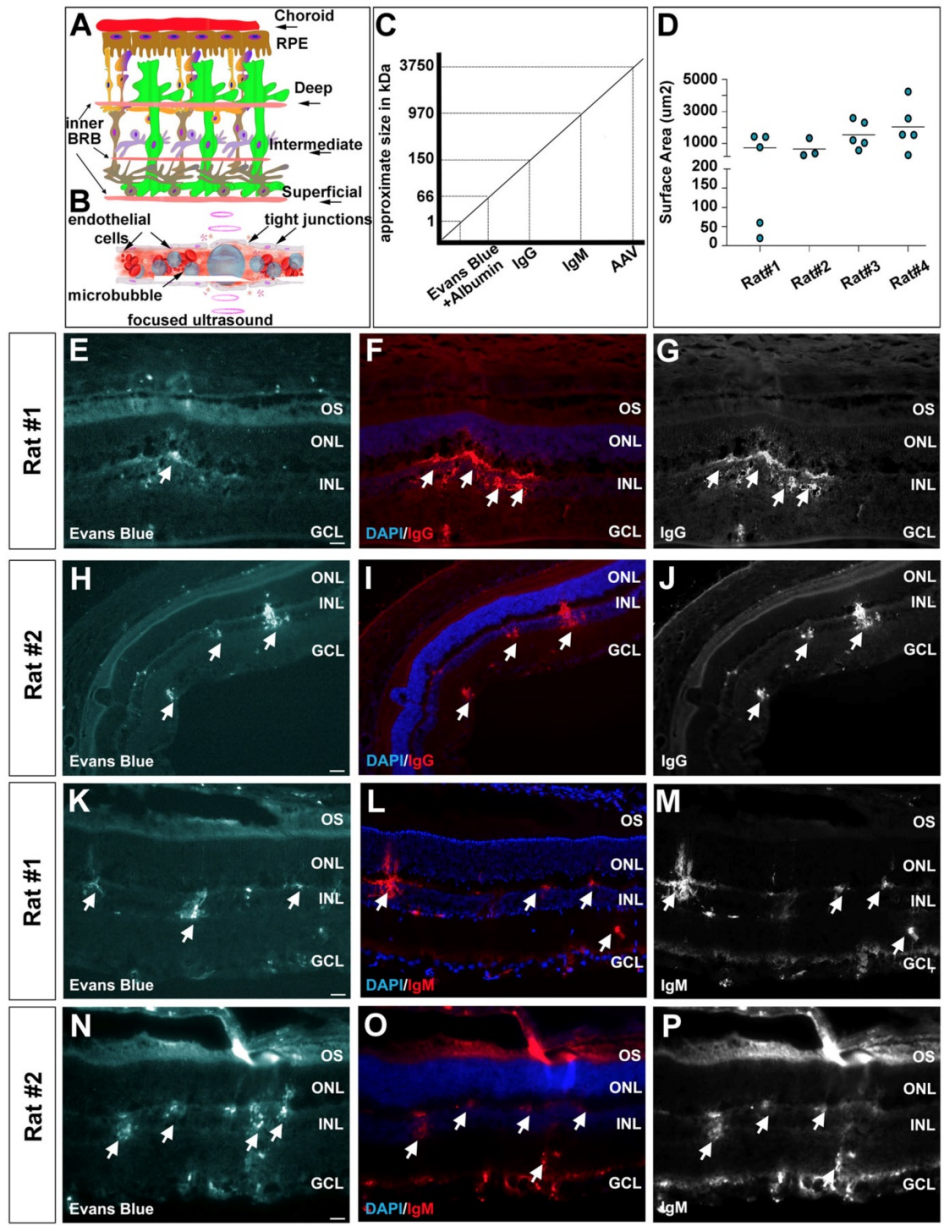
** FUS permeabilization of the blood-retinal barrier as revealed by the entry of macromolecules into the retinal parenchyma.** (A-C) Schematic illustration of the retinal vasculature, including the deep, intermediate and superficial vessels that infiltrate the retina and form the inner BRB, and the choroid that overlies the RPE (outer BRB) (A). Schematic illustration of the effects of focused ultrasound on microbubbles, as they capture acoustic energy and permeabilize the tight junctions that make up the BRB (B). Schematic illustration of the sizes of the different molecules that we tested for entry into the retinal parenchyma after FUS (C). (D) Surface area quantification of Evans blue per section of 4 positive rats. (E-P) Photomicrographs showing entry of Evans blue (arrow heads) (E,H,K,N), IgG (F,G,I,J) and IgM (L,M,O,P) into the retinal parenchyma of two representative rats. Blue is nuclear DAPI counterstain in E,H,K,N. GCL, ganglion cell layer; INL, inner nuclear layer; ONL, outer nuclear layer; OS, outer segments. (Scale bars=25 μm).

To further test BRB integrity after FUS, we used an Evans blue permeability assay; injecting the Evans blue dye immediately after microbubble injection and FUS, and harvesting rat eyes 30 min later for sectioning. Evans blue binds to plasma albumin, and transportation into the retina is indicative of protein leakage across the BRB 59. In particular, we asked whether there was leakage through the superficial, intermediate and deep vascular plexi (inner BRB) or the choroid (lying above the RPE, which is the outer BRB) based on the vicinity of dye uptake (Fig. 4A). Microbubbles capture acoustic energy and they respond by contracting and expending, causing pressure and mechanical stresses on the vessel walls, and leading to the transient perturbation of tight junctions between endothelial cells (Fig. 4B). In five out of six rats tested, we observed Evans blue dye uptake in the retina, including most prominently in the INL and ganglion cell layer, suggesting that the vascular plexi within these layers were indeed permeabilized (examples from two rats shown in Fig. 4E,H,K,N). The quantification of Evans blue surface area in 4 rats showed an average opening of 744.2, 675.0, 1554 and 2046 μm² per section (Fig. 4D). In contrast, there was little evidence that Evans blue passed through the choroid and into the retina (Fig. 4E,H,K,N), suggesting that this route may not be accessible with FUS.

Evans blue dye has a relatively small size (0.96 kDa), even when bound to albumin (66 kDa), especially compared to AAV particles, which are 3746 kDa in mass, or ~25 nm in diameter (Fig. 4C). To test the extent to which the BRB was permeabilized we examined whether larger, blood-borne molecules, were transmitted across the barrier. Specifically, we focused on the monomer immunoglobulin IgG (150 kDa), and pentamer immunoglobulin IgM (970 kDa) (Fig. 4C). In all five rats in which Evans blue dye uptake was observed, we also detected IgG (Fig. 4F,G,I,J) and IgM (Fig. 4L,M,O,P) molecules in the retinal parenchyma. Again, the uptake of these macromolecules was most prominent in the vicinity of the deep and superficial vascular plexi, with no evidence of transfer across the choroid and into the RPE.

In sum, our data reveal that FUS effectively targets the vascular plexi that infiltrate the retina, increasing the permeability of the inner BRB and allowing the transfer of macromolecules into inner retinal cells.

### Minimal evidence of retinal damage following FUS

**Figure 5:**
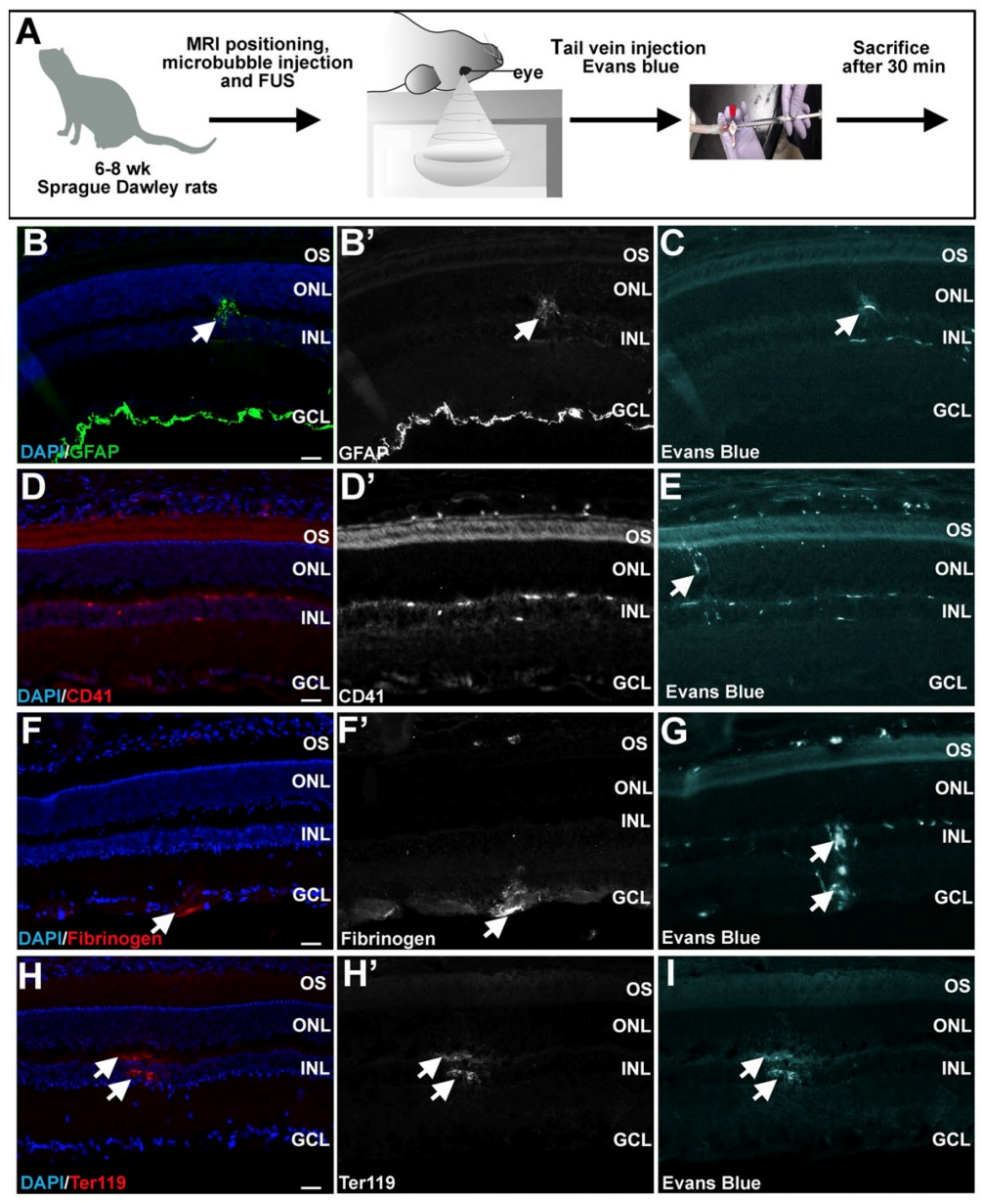
** Minimal retinal damage observed 30 minutes after FUS.** (A) Schematic illustration of FUS procedure in 6-8 week old Sprague Dawley rats. (B-I) Representative photomicrographs showing expression of GFAP (arrowheads, B,B'), CD41 (D,D'), fibrinogen (arrowheads, F,F') and Ter119 (arrowheads H,H') in the retinal parenchyma in the vicinity of areas where Evans blue permeabilization was evident (C,E,G,I). GCL, ganglion cell layer; INL, inner nuclear layer; ONL, outer nuclear layer; OS, outer segments. (Scale bars=25 μm).

Upon successful BRB permeabilization, we tested whether FUS caused severe glial activation and damage to the rat retina (Fig. 5A). We first investigated potential evidence of reactive gliosis, which is a neuroprotective injury response that Müller glia undergo, resulting in an upregulation in expression of intermediate filament proteins such as GFAP 3. Screening for reactive gliosis is important, because while moderate gliosis is transient and protective (and in this study allows mCherry expression from the GFAP promoter), severe gliosis is cytotoxic, and can scar and remodel the retina permanently 3. Immunofluorescence staining was carried out for GFAP on all six retinas harvested 30 min after FUS, and only observed to be elevated in one out of six experimental rats, correlating with the site of Evans blue permeabilization (Fig 5B,B',C). This data suggests that the damage response is not overtly activated in retinas immediately post-FUS, although GFAP expression is evident at later stages (see gene delivery below).

Another way to assess damage is to test for CD41 expression marking megakaryocyte infiltration. CD41 expression was not observed in any experimental rats, even in the vicinity of Evans blue uptake (Fig. 5D,D',E). We also asked whether fibrinogen, which is a serum glycoprotein, passes into the retina, as its presence is not only associated with BBB disruption, but it also plays a role in neuroinflammation 60. Fibrinogen was observed in two out of six retinas exposed to FUS, overlapping with the site of Evans blue infiltration (Fig. 5F,F',G). Similarly, Ter119, which marks erythroid cells, was detected in two out of six of the FUS-treated retinas, again overlapping with Evans blue uptake (Fig. 5H,H',I). To further analyze potential damage of FUS to retinal stucture and morphology, we examined histology. An analysis of hematoxylin and eosin stained or DAPI stained FUS-treated or control retinas revealed no morphological damage, except in one of six rats (Fig. S1A-D).

In summary, while the presence of fibrinogen and Ter119 staining in FUS-treated retinas suggests that FUS does have the potential to open the retinal vasculature to an extent that cells in the blood may pass into the retinal parenchyma, there is little evidence of a damage response in the retina, given that CD41 megakaryocyte invasion was not observed, GFAP upregulation was limited, at least in the immediate aftermath, and morphological disruptions were not very frequent (17% of rats).

### Systemic delivery of AAV2/8-hGFAP-mCherry to retinal glial cells with focused ultrasound

**Figure 6:**
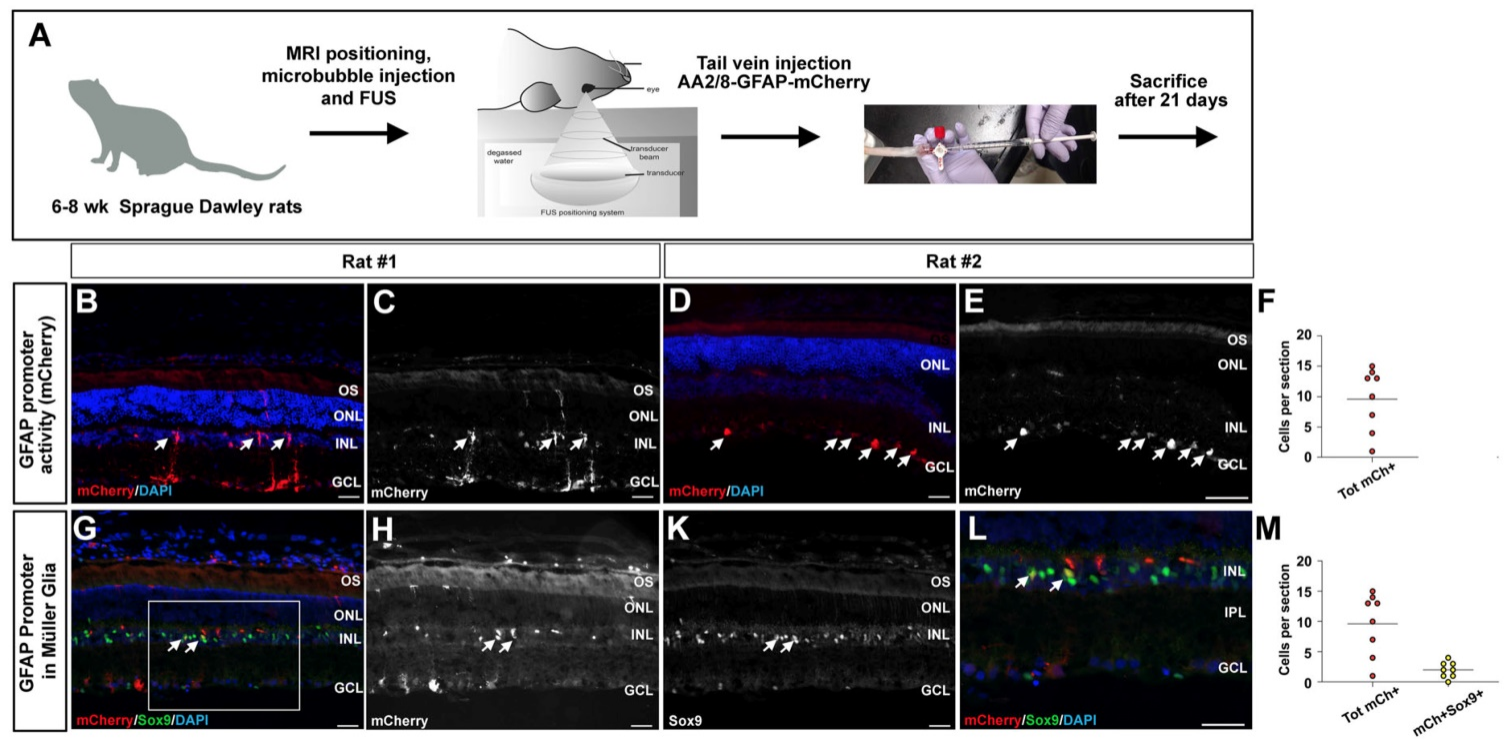
** FUS delivery of AAV2/8-GFAP-mCherry into the retina.** (A) Schematic illustration of experimental procedure, showing tail vein catheter used to deliver microbubbles, then FUS, and then AAV2/8-GFAP-mCherry. Animals were then harvested 21 days later. (B-E, G-L) mCherry epifluorescence in Müller glia-like cells in the inner nuclear layer (arrows, B,C). (G-L) Co-labeling of mCherry and Sox9 in Müller glia in the inner nuclear layer (arrows, G-L). Blue is nuclear DAPI stain. Boxed areas in Gis magnified in L. (F,M) Quantification of total mCherry cells (F) and mCherry+Sox9+ cells per section (M). GCL, ganglion cell layer; INL, inner nuclear layer; IPL, inner plexiform layer; ONL, outer nuclear layer; OS, outer segments. (Scale bars=25 μm).

Our next goal was to evaluate the efficacy of FUS to deliver AAV2/8-hGFAP-mCherry into rat Müller glia by permeabilizing the inner vascular plexi of the retina. Tail vein catheters were inserted into three rats for delivery of the virus immediately after FUS and microbubble injections (Fig. 6A). We tested two concentrations of AAV2/8-hGFAP-mCherry; 2.5×10^9^ GC/g, and 1.25×10^8^ GC/g, with a stock virus of 2.25x10^12^ GC/ml diluted to 500 μl injected per animal (based on body weight of ~300 g). Animals were sacrificed after three weeks, and retinas were sectioned and analyzed for mCherry expression. Notably, no macroscopic abnormalities were observed in the retinas upon dissection, including a lack of lens abnormalities (i.e., cataracts).

We observed transduced cells expressing mCherry only in rats that received the higher viral concentration (2.5×10^9^ GC/g), and only in the experimental eyes where FUS was applied were mCherry positive cells detected (Fig. 6B-E), with an average of 10 cells per section (Fig. 6F). In these rats, mCherry was expressed in Müller glia-like cells that had their cell bodies in the INL and extended processes to the ILM and OLM (Fig. 6B,C), as well as in astrocyte-like cells underlying the GCL, in the nerve fiber layer (Fig. 6D,E). To confirm the identities of these transduced cells, we performed co-immunolabeling with mCherry and Sox9 to label Müller glia and mCherry and Aldolase for astrocytes. In retinas that had mCherry expression in the nerve fiber layer, co-labeling was observed with Aldolase, affirming that the transduced cells were astrocytes (Fig. S2A-D). In addition, in retinas with radially projecting cells in the INL, co-labeling was observed with Sox9, confirming that the transduced cells were indeed Müller glia (Fig. 6J-M) with 20% of total mCherry+ cells expressing Sox9 (Fig. 6L).

These results demonstrate that following the systemic administration of AAV2/8-hGFAP-mCherry together with microbubbles and FUS, both rat retinal astrocytes and Müller cells can be successfully transduced.

### GFAP promoter narrows down peripheral expression to liver and kidney

**Figure 7:**
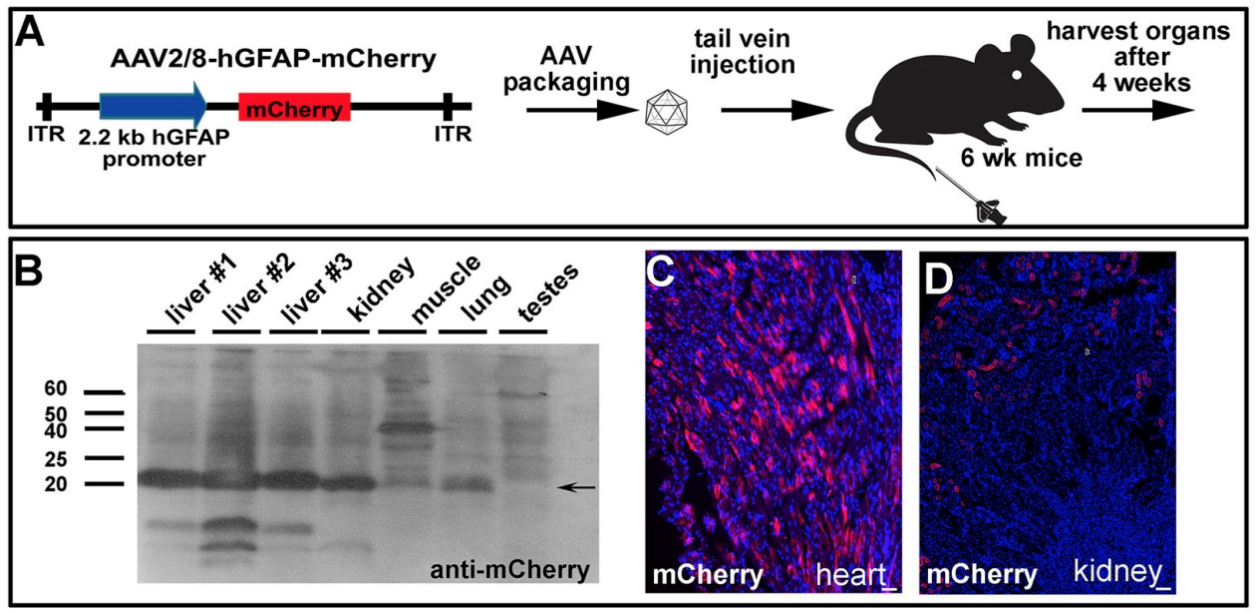
** Systemically injected AAV2/8-GFAP-mCherry is trapped and expressed in peripheral organs.** (A) Experimental procedure, showing AAV2/8-GFAP-mCherry construct and tail vein injection in 6 week old mice. (B) Western blot of lysed organs with an anti-mCherry antibody, confirming expression of this fluorescent protein in liver, kidney and lung 4 weeks post injection. (C) mCherry epifluorescence in the heart (C) and kidney (D). (Scale bars =25 μm).

An important consideration for gene therapy, especially for therapeutics delivered systemically, is that cells in the periphery may also be transduced and express the transgene. This issue is particularly important given that organs outside of the CNS do not have the same barriers to the bloodstream. We therefore set up an experiment in wildtype C57Bl/6 mice to determine whether AAV2/8-hGFAP-mCherry delivered into the tail vein was taken up and expressed by any peripheral organs (Fig. 7A). Four weeks post systemic injection, we harvested quadricep muscle, testes, kidney, heart, lung and liver for biochemical analyses (Fig. 7B), as well as fixed tissues for immunohistochemical studies (Fig. 7C,D). Notably, the liver was visibly a darker red in all three animals compared to controls, suggesting high levels of mCherry expression (not shown). To test whether mCherry was indeed expressed, tissues were lysed and run on SDS-PAGE gels, and proteins were then transferred to PVDF membranes for Western blotting. We observed a very strong immunoreactive band when the membranes were blotted with anti-mCherry in all three livers from each of the animals, as well as in the kidney, and to a lesser extent in the muscle, and lung, but not in the testes (Fig. 7B). We also observed mCherry immunoreactive cells in tissue sections, including in the heart (Fig. 7C) and kidney (Fig. 7D).

Our data demonstrate that FUS can be used for the delivery of AAV2/8, administered intravenously, to the retina. AAV2/8-hGFAP-mCherry was delivered with FUS and microbubbules to rat astrocytes and Müller cells, achieving glial specificity in the retina. However, the current hGFAP promoter was also active in peripheral organs, which the AAV2/8 vector has tropism for.

## Discussion

The goal of this study was to identify a viral vector and delivery strategy that could be used to transduce retinal Müller glia, a cell type that has therapeutic potential 61. We first tested the systemic delivery of AAV-PHP.eB, which had previously been shown to traverse the BBB and BRB 37, 40, but we did not observe transduction of Müller glia. While intravitreal injections of various AAVs have been demonstrated to successfully target Müller glia 25, 26, bypassing the inner limiting membrane, given the potential for damage with this delivery route, we asked whether FUS could be used as a less invasive strategy to target this cell population. We found that FUS combined with microbubbles can increase cell membrane permeability of the inner BRB, allowing for the passage of macromolecules (Evans blue-albumin, IgG, IgM) and viruses (AAV2/8) into the retinal parenchyma.

FUS-induced BBB 27 and BRB 36 permeabilization has been shown previously to be transient, with the integrity of CNS barriers being restored rapidly, making it safe and effective for gene delivery. In the retina, a prior FUS study showed that the BRB closes within 3hr post-FUS 36. Based on evidence that BBB permeability is greatest immediately post-FUS 27, 62, the administration of the virus together with, or immediately after, intravenous microbubble delivery and ultrasound pulses, is also likely to be the most effective strategy for gene delivery to the retina. The transient nature and safety of FUS has been well established in the brain, and indeed, has now even been shown to induce a transient opening of the BBB in patients with Alzheimer's disease 63 and amyotrophic lateral sclerosis 30, with closure confirmed 24 hr post-operation. The FUS procedure also results in minmal damage to the eye; of the six rats we performed FUS on for our permeability assays, only one demonstrated a morphological disruption post-FUS. We also observed minimal red blood cell extravasation, and no macrophage/microglia infiltration. While studies in the brain have suggested that there is an inflammatory response when FUS is applied to the brain 64, this response is transitory 65, and the immune response can also be beneficial to repair in the short-term.

Ultimately our goal is to use FUS to deliver gene therapies to stimulate the repair potential of Müller glia in degenerative diseases. One question is whether FUS will be required to permeabilize the inner BRB to deliver gene therapies in degenerative diseases associated with a 'leaky' vasculature, including retinal proliferative ischemic retinopathies 66 and wet AMD 67. In these conditions, angiogenesis occurs in an uncontrolled fashion, but the newly formed vessels are abnormal and not tightly sealed, which can result in vision loss if the macula is affected. One reason why it still may be important to use FUS to permeabilize the inner BRB for gene transfer is that new vessels form randomly and sporadically in these diseases, and their presence in required areas cannot be relied upon for gene transfer. Another reason is that abnormal vessels can be efficiently prevented/repaired by different drugs, primarily including anti-VEGF agents, as part of palliative therapies 68. Thus, while it will be important to test the utility of using FUS in a model of AMD, the advantage of FUS for targeted gene delivery is that it will provide a transitory and controlled permeabilization of intact vessels.

The use of ultrasound for macromolecular delivery in retinal cells has been demonstrated before, including antibody delivery in RPE and Müller glial cell lines *in vitro* using nanobubbles and ultrasound 69. Prior to our study, a few groups had also demonstrated that the intravitreal delivery of AAVs to the retina could be enhanced when combined with FUS 70, 71. However, in one of these studies, the US transducer was directly transplanted in the rabbit vitreous, and the authors used plasmid DNA, necessitating the transient opening of cellular membranes for gene transfer, and hence, a more disruptive ultrasound force 71. In the second study, high intensity ultrasound was used to deliver AAVs to retinal ganglion cells, relying on microbubble destruction to open cell membranes after intravitreal injection 70. While these studies provided a proof of principle for using FUS in retinal gene delivery in rats and larger animals (rabbits), both techniques are more invasive than the low frequency ultrasound used in our study, and as such, these earlier approaches are unlikely to be suitable for future human applications.

One issue with FUS is that the ultrasound passes through the retina and thus can target other cells in the path. Indeed, in addition to demonstrating that we could effectively deliver AAV2/8-GFAP-mCherry to Müller glia, we also transduced astrocytes in the nerve fiber layer. The systemic delivery of AAVs followed by FUS could also facilitate delivery across the outer BRB, potentially delivering AAVs across the RPE and into the outer nuclear layer (ONL), where photoreceptors reside. While we did not observe Evans blue in the RPE or ONL, future studies would need to look at this more carefully, using an AAV construct with a capsid-promoter combination that would target photoreceptor cells. If the RPE and ONL are targeted via FUS, implying that this technique could open up the choroidal vessels, then it would be possible to control for the specificity of cell transduction in other ways. Indeed, cells that express the DNA cargo in AAV-based gene therapy vectors depends not only on the route of delivery, but also on the AAV capsids, which have tropism for different cell types, and promoter/regulatory elements, which drive gene expression in specific cell types. In our study, we demonstrated that the AAV2/8-GFAP-mCherry vector is glial specific, selectively driving reporter gene expression in Müller glia in the retina (and in astrocytes in the nerve fiber layer and brain). There should thus be no consequence of uptake of the viral vector by neuronal cell types in the retina.

We would like to highlight that the AAV must be injected immediately after or together with the microbubbles, followed by FUS application so that the virus enters the circulation when the BRB is best permeabilized. In contrast, injecting the virus first, and then applying FUS at different time points, would allow the virus to circulate through the body and have an increased opportunity to transduce peripheral organs, which do not have barriers with the blood, resulting in viral titer dropping over time. Indeed, we and others 72, have observed a transduction of peripheral organs with AAV2/8-GFAP-mCherry after systemic delivery, which is required for combining with FUS. This result is not surprising given that the barriers between the blood and CNS tissues do not exist in other organs. Moreover, GFAP expression has been reported in several non-neural tissues, including hepatic stellate cells in fibrotic livers 73, 74, in skeletal muscle, especially in certain disease conditions, which may be contaminated by peripheral nerve Schwann cells that innervate these muscles 75, in cardiac fibroblasts 76 and in kidney podocytes 77. Moving AAV-based therapies to the clinic will require that strategies to limit peripheral organ expression are found. One way to move forward would be to combine different AAVs, such as AAV-ShH10, which is related to AAV6 and has a strong tropism for Müller glia 21, 44, 45, with Müller glia-specific promoter sequences, which together may minimize peripheral organ expression. Such an approach will require that the safety of AAV-ShH10 is further tested in pre-clinical models before it can move to clinical trials. In addition to the GFAP promoter, other promoters have been used to drive gene expression in Müller glia, such as the CD44, vimentin and Rlbp1 promoters 24, 78, 79. Another potential source of new promoter elements may come from the Pleiades Promoter Project that is based on computational biology and phylogenetic conservation of source genes, resulting in the creation of at least two artificial 'mini-promoters' that drive Müller glia expression (Ple25 and Ple53) 80. However, these promoters are each ~ 3 kb in size, not leaving much room in the 4.7kb packaging limit of AAVs for target genes. In addition, both of these promoters are also expressed in other retinal cells, including retinal interneurons and ganglion cells 80. A comprehensive analysis of transduction levels in peripheral organs with these constructs at different doses would help us to identify the best AAV capsid/promoter combinations for therapeutic delivery to retinal Müller glia.

In summary, the ability to deliver gene therapies to Müller cells so as to 'activate' their stem cell properties has great potential for retinal repair. Several rate-limiting factors have been discovered that regulate Müller glia proliferation and differentiation. 6-8, 81-89. Whether it be gene or cell-based therapies that are ultimately adapted in the clinic, the delivery of these therapeutics across the BRB is a rate-limiting factor that must be optimized as we move forward.

## Conclusions

Our findings are important for several reasons. Firstly, we revealed that the inner vascular plexi in the retina can be permeabilized by FUS in the presence of microbubbles to an extent that allows for the delivery of AAV particles, which are in the size range of ~ 25 nm. Our study is the first to demonstrate viral delivery across the inner BRB, as other studies targeting Müller glia have all used intravitreal injections that bypass the inner limiting membrane. Secondly, we demonstrated that the combination of AAV2/8 with a GFAP promoter has tropism for cortical and retinal glial cells. Future work will explore options to minimize peripheral organ transduction by AAV2/8, allowing FUS to be a safe and non-invasive methodology suitable for clinical gene therapy for several CNS neurodegenerative disorders. Moreover, given our finding that ~17% of animals displayed a morphological disruption immediately post-FUS, future studies would be required to optimize FUS parameters to limit this damage. Ideally, retinal safety assessments testing the feasibility of using FUS technology to safely deliver AAVs to the inner retina would be performed longitudinally on live animals using optical coherence tomography (OCT) to study retinal morphology and electroretinograms (ERG) to study retinal function.

## Methods

**Animals.** All animal procedures were approved by the Sunnybrook Research Institute Animal Care Committee (ACC Protocol # 19627 and # 17614) in agreement with the Guidelines of the Canadian Council of Animal Care (CCAC). All *in vivo* experiments were performed on 6-8-week-old Sprague Dawley rats, C57BL/6 mice, hybrid C3H/He-C57BL/6 mice and CD1 mice, as indicated (all from Charles River, Sherbrooke, QC, Canada). Rats and mice were housed in cages under 12:12 hour light/dark cycles. All animals used in this study are summarized in Table S2.

**Tissue collection, processing and sectioning.** Prior to sacrifice, rats and mice were anesthetized with ketamine and xylazine (75 and 10 mg/kg, respectively) and then perfused transcardially, with 0.9% saline and 4% paraformaldehyde (PFA). Brains were collected and post-fixed overnight in 4% PFA followed by transfer into 30% sucrose overnight, followed by sectioning of 40 μm horizontal sections using a Leica SM2010r microtome (Leica Microsystems Canada Inc., Richmond Hill, Ontario, Canada). Eyes were dissected after perfusion and post-fixed overnight in 30% sucrose at 4°C. After embedding in O.C.T. compound (Tissue-Tek® O.C.T. Compound, Sakura® Finetek, PA, USA), retinal sections were cut transversally (12 μm) on a Leica CM3050 cryostat (Leica Microsystems Canada Inc., Richmond Hill, Ontario, Canada).

**Systemic injection of AAV-PHP.eB in mice.** 7-month-old hybrid C3H/He-C57BL/6 female mice were injected intravenously with 3.33x10^9^ GC/g AAV-PHP.eB (Addgene, ID: 37825; Watertown, MA, USA) in 200 μL using an insulin syringe. Mice were heated for 30 min in their cages under a blanket using a bearhugger to increase visibility of the tail veins. Mice were anaesthetized with 5% isoflurane and kept under 2-3% isoflurane during tail vein injections.

**AAV2/8 transduction of mouse retinal explants.** Postnatal day (P) 3 mouse eyes were enucleated, and the retina was dissected in ice-cold PBS, removing the lens, RPE and cornea. Cup-shaped retinas were flattened with four cuts and placed on 0.25-μm Nucleopore® track-etched membranes. The membrane was then floated on retinal explant medium (50% Dulbecco's Modified Eagle Medium [DMEM], 25% Hanks' Balanced Salt Solution [HBSS], 25% heat inactivated horse serum, 200 μM L-Glutamine, 0.6 mM HEPES, 1% Penicillin/Streptomycin, 0.2% amphotericin B). 1.5 μl of 1.25×10^8^ GC/ml AAV2/8-GFAP-mCherry was pipetted onto the top of each explant. Explants were then cultured at 37^o^C in 5% CO_2_ for 7 days *in vitro* (DIV). Retinal explants were then rinsed with PBS before fixation in 4% PFA in 1xPBS O/N at 4^o^C. The fixed retinas were then blocked and sectioned as described.

**Intracranial injection of AAV2/8 into mouse brain.** 9 week old C57BL/6 mice were anaesthetized by inhalation anesthesia with isoflurane (2%, 1L/min with medical air). Intracranial injections were performed using a stereotax and bregma and lambda levels were adjusted, followed by drilling through the skull and the injection of 1.0x10^9^ GC in a 1 uL total volume at 0.1µl/min over the span of 10 mins. We injected in the right and left hemisphere with the following coordinates: AP 1.41 mm, ML +1.75 mm, DV - 1.7mm from the skull. 21 days later, animals were sacrificed and tissues were harvested as described.

**Intravitreal delivery of AAV2/8 in rats.** For intravitreal delivery, Sprague Dawley rats were anaesthetized by inhalation anesthesia with isoflurane (2%, 1L/min with medical air). A small incision was carefully made close to the limbus with a 30-gauge needle, then 2 μL of AAV2/8-GFAP-mCherry at 2×10^12^ GC/ml was introduced into the vitreous using a pulled glass needle controlled by a FemtoJet (Eppendorf Canada Ltd. Mississauga, ON, Canada) in each eye. Following the intravitreal injection, the eye was covered with 0.3% hypromellose gel (GenTeal Lubricant Eye Gel, Alcon Canada). Animals were sacrificed 21 days after injection as described.

**Magnetic resonance imaging in rats.** 6-7 week old Sprague Dawley rats were anesthetized with isoflurane (5%, 1L/min with medical air). Hair around the eye was removed with a depilatory cream (Nair™, Church and Dwight Canada Corp., Mississauga, ON, Canada). A 22G angiocatheter was placed into the tail vein. The eye was protected with 0.3% hypromellose gel (GenTeal Lubricant Eye Gel, Alcon Canada, Mississauga, ON, Canada). A nose cone was used to maintain 2% isoflurane during the MRI-FUS procedures. The animal was placed on a removable sled in the treatment position and the sled was then positioned in the MRI scanner and imaging performed. T1- and T2- weighted FSE images were obtained before and after the administration of gadolinium into the tail vein, and used to identify focal regions for FUS administration (asterisks in D',H',L',P'). MR images have a resolution of 0.3x0.3 mm^2^ in the x/y-plane and 1 mm in the z-plane, which is sufficient to detect focused ultrasound spots of approximately 0.8 mm in diameter and 5 mm in length.

**Locating focal regions of FUS.** Prior to the experiments the focus of the ultrasound transducer was localized by placing a small container of water on the sonication sled with the water surface at the focal depth of the transducer. Then the ultrasound transducer was turned on at a low power resulting in a small fountain at the focus location. A MRI visible marker was placed on the location of the fountain and the MR imaging repeated. The location of the marker in the images registered the image space in the sonication space. This method has been described and was found to be reproducible in an earlier study in the brain 57.

**Synchronization of MRI and FUS device.** The focused ultrasound system used is MR imaging-compatible and operationally similar to the system we previously described 56. However, due to the small bore size of the 7T magnet, sonications were performed outside of the magnet as described 57. After MR imaging, the sled was placed on the sonication system and the MR images were used to target sonication locations. After sonication, the animal was reimaged to verify BRB modulation and its location.

**Verification of bubble effect when FUS is on.** The effect of the ultrasound on the microbubbles was verified by monitoring acoustic emission from the exposed microbubbles using a hydrophone located in the middle of the sonication transducer. The pressure was gradually increased in each burst until sub-harmonic emissions were detected and then continued at 50% of the scaling level. This method has previously been shown to induce a post-sonication enhancement in gadolinium in about 19% of rat brains 58.

**Focused ultrasound procedure and gadolinium and Evans blue permeability assays.** After MR imaging, the sled was placed on the sonication system (Figure 3A-C). Definity H microbubbles (Lantheus Medical Imaging Canada, Mississauga, ON, Canada) of 1.1 - 3.3 μm diameter were delivered through a tail vein catheter at 0.2 ml/kg (1:9 ratio Definity: saline) followed by a saline flush (0.5 ml). A set burst of sonication was applied through the cornea using an in-house constructed prototype device (similar in operation to LP-100, FUS Instruments, Toronto, ON, Canada) with a focused transducer (diameter 70 mm, radius of curvature of 60 mm, frequency of 1.1 MHz). The pressure amplitude of 10 ms sonications (repetition frequency of 1Hz) was first ramped up until sub-harmonic emissions were detected by a hydrophone and then sonications were continued at 50% of the maximum pressure amplitude 58 for a total sonication time of 120s. This resulted in a pressure range of 0.360 - 0.84 MPa after the 50% drop (see table.S1 for summary).

The hydrophone was in-house manufactured with an active element diameter of 2.5mm (area 4.9mm^2) (Material: DL-47DeL Piezo Specialities, LLC, USA) and an approximate bandwidth of 33%. As a single small detector it was sensitive to all events in the ultrasound field. Since in these experiments the pressure amplitude was gradually increased it was assumed that the threshold event would first appear at the focus where the pressure amplitude was the highest. The cavitation events could not be spatially resolved within in the focus. The time average acoustic powers are less than 5 mW, thus eliminating the possibility of any detectable temperature rise 2. After sonication, serial contrast enhanced T1- weighted FSE images were obtained at 1 min, 4 min, 5 min, 10 min, and 15 min to look for uptake of contrast media in the retina, verifying the location of BRB modulation (Fig. 3F,F',J,J',N,N',R,R'). For the Evans blue permeability assay, immediately after sonication, each animal was injected with 4 mg/ml/Kg body weight of 0.5% Evans blue (Sigma Aldrich Canada, Mississauga, ON, Canada), diluted in phosphate buffered saline (PBS) and filter sterilized. Animals were sacrificed 30 min later as described.

**FUS delivery of AAV in rats.** The AAV2/8-GFAP-mCherry construct was previously described 46 and was sent for packaging and ultra-purification to VectorBuilder Inc (Chicago, Illinois, USA). The AAV2/8-GFAP-mCherry stock (2.25× 10^12^GC/ml) was diluted in PBS to a final volume of 500 μl to deliver two different amounts; 2.5×10^9^ GC/g and 1.25×10^8^ GC/g, with the final concentration calculated based on an average weight of 300 g/rat. For FUS delivery, 0.5 ml of the diluted virus was injected through a tail vein catheter immediately after the microbubbles were administered, followed by a saline flush (N=2 animals). Animals were sacrificed 21 days later as described.

**Tail vein AAV injection in mice for peripheral organ harvesting.** Three adult C57BL/6 mice received tail vein injections of AAV2/8-GFAP-mCherry at one of two concentrations; 2.5×10^9^ GC/ml (low) or 1.25×10^10^ GC/ml (high). Briefly, mice were heated under a blanket using a bair hugger for 30 min to increase visibility of tail-veins prior to injection. AAV was diluted in PBS to a total volume of 200 uL for each mouse and injected in the tail-vein of isoflurane anesthetized mice. Four weeks later, mice were sacrificed as described.

**Immunohistochemistry.** Sections were blocked 1 hour at room temperature in PBS with 10% donkey or horse serum, with or without 3% bovine serum albumin and 0.1-0.3% Triton-X-100, and then incubated overnight at 4°C in primary antibodies diluted in blocking solution, including rabbit anti-Sox9 (1:500, #AB5535, Millipore Canada Inc, Etobicoke, Ontario, Canada), rat anti-mCherry (1:500, # M11217, Invitrogen Canada Inc., Burlington, Ontario, Canada), rabbit anti-GFP (1:1000, #AB3080, Millipore Canada Inc, Etobicoke, Ontario, Canada), goat anti-Aldolase (1:500, #Sc-1206, Santa Cruz Biotechnology, Inc., Mississauga, Ontario, Canada), rabbit anti-GFAP (1:750, #G9269, Sigma Aldrich Canada, Mississauga, ON, Canada), rabbit anti-fibrinogen (1:500, #A0080, Dako Canada Inc., Burlington, Ontario, Canada), rabbit anti-CD41 (1:100, #ab63983, Abcam, Cambridge, MA, USA), and mouse anti-TER119 (1:100, #553671, BD Biosciences, Mississauga, Ontario, Canada). Slides were washed three times in PBS with 0.1% triton X-100 (PBT) and incubated with species-specific secondary antibodies conjugated to Alexa568 (1:500; Invitrogen Molecular Probes™, ThermoFisher Scientific, Markham, ON, Canada), Alexa488 (1:500; Molecular Probes) or Cy3 (Abcam, Cambridge, MA, USA) for 1 hour in room temperature. Slides were then washed three times and counterstained with 4,6-Diamidino-2-phenylindole (DAPI) (Sigma Aldrich Canada, Mississauga, ON, Canada). Finally, the slides were washed three times and mounted in Aqua-polymount (Polysciences Inc., PA, USA).

**Fluorescence Imaging.** Images of AAV-PHP.eB transduced brains were acquired using an AxioScan.Z1 (Carl Zeiss Canada Ltd. North York, Ontario, Canada). All other images were either captured with a fluorescence microscope (DM RXA2) (Leica Microsystems Canada Inc., Richmond Hill, Ontario, Canada) or Nikon A1Confocal. The images were prepared using Adobe Photoshop CC 2017 (Adobe Systems Inc., San Jose, CA, USA).

**Western blotting.** Harvested organs were lysed in RIPA buffer (1% triton-100, 1% sodium deoxycholate, 0.1%SDS in PBS) with protease inhibitors (1 Complete EDTA-free Tablet resuspended in 4 ml water; Hoffmann-La Roche Ltd., Mississauga, ON, Canada) and 1mM Phenylmethanesulfonyl fluoride (PMSF) (Sigma Aldrich Canada, Mississauga, ON, Canada). Samples were placed on ice for 5 min, before spinning at 4 °C for 10 min at 16,000 rcf. 20 μg of lysate was mixed in SDS-PAGE loading dye (buffer (100 mM Tris-HCl [pH 6.8], 25% glycerol, 2% SDS, 0.01% Bromophenol Blue, 10% β-mercaptoethanol), denatured at 95ºC for 5 min, and loaded on a 10% SDS-polyacrylamide gel that was run at 124 V until the dye front reached the end of the gel. Protein was transferred to a PVDF membrane (Bio-Rad Laboratories, Mississauga, ON, Canada) at 45 V at 4 ^o^C using a transblot apparatus (Bio-Rad Laboratories). Membranes were blocked in 5% skim milk powder in TBST (tris-buffered saline [TBS], pH 7.5 with 0.1% tween 20). Blots were then incubated at 4 °C overnight in anti-mCherry antibody (#AB0040-200, SICGEN - Research and Development in Biotechnology Ltd. Cantanhede, Portugal) diluted in TBST (1:10,000 dilution). Blots were washed in TBST and incubated in secondary rabbit-α-Goat IgG HRP (1:10,000 dilution) (BioRad #172-1034) for 1 hour at room temperature. Blots were washed again with TBST and developed with ECL Plus (RPN2236, GE Healthcare, Mississauga, ON, Canada) and exposed to light sensitive X-ray film (Kodak/Fujifilm, Mississauga, ON).

**Quantification.** All statistical analysis were conducted using GraphPad Prism Software version 8.3.0 (GraphPad Software). Error bars represent standard error of the mean (s.e.m.). Cells were counted on a minimum of three eyes per condition and a minimum of three sections per eye. Gadolinium enhancement was quantified by measuring the integrated density of ROI using ImageJ. Evans Blue surface area was quantified using the surface area measurement in ImageJ then pixels were converted into µm^2^ according to the corresponding scale bar.

## Supplementary Material

Supplementary figures and tables.

## Abbreviations

AAV: adeno-associated virus
AMD: age-related macular degeneration
BBB: blood-brain-barrier
BRB: blood-retinal barrier
CNS: central nervous system
FUS: focused ultrasound
GC: genome copies
GCL: ganglion cell layer
GFAP: glial fibrillary acidic protein
ILM: inner limiting membrane
INL: inner nuclear layer
MRI: magnetic resonance imaging
OLM: outer limiting membrane
ONL: outer nuclear layer
P: postnatal day
PBS: phosphate-buffered saline
PFA: paraformaldehyde
RP: retinitis pigmentosa
RPE: retinal pigment epithelium
