# Supplementary material for: Focused ultrasound as a novel strategy for noninvasive gene delivery to retinal Müller glia
Source: Theranostics. 2020 Feb 10;10(7):2982–99. doi: 10.7150/thno.42611 (PMC7053200; doi:10.7150/thno.42611)
Supplement: Supplementary file 1 — Supplementary figures and tables. [file thnov10p2982s1.pdf]

Supplemental Figure S1. FUS disrupted retinal morphology in one of six rat eyes. (A,B) DAPI (A,B) and Hematoxylin and Eosin (C,D) staining of rat retinas 30 minutes after FUS (B,D) and untreated controls (A,C). Red arrowheads in B,D point to sites of retinal dysmorphology. (Scale bars =25 μm).

Supplemental Figure S2. FUS delivery of AAV2/8-GFAP-mCherry into retinal astrocytes. (A-D) mCherry epifluorescence in astrocyte-like cells in the nerve fiber layer (arrows, A-C). (A, C) Co-labeling of mCherry and aldolase in astrocytes in the nerve fiber layer (arrows, A-C). Blue is nuclear DAPI stain. Boxed areas in A is magnified in D. GCL, ganglion cell layer; INL, inner nuclear layer; IPL, inner plexiform layer; ONL, outer nuclear layer; OS, outer segments. (Scale bars=25 μm).

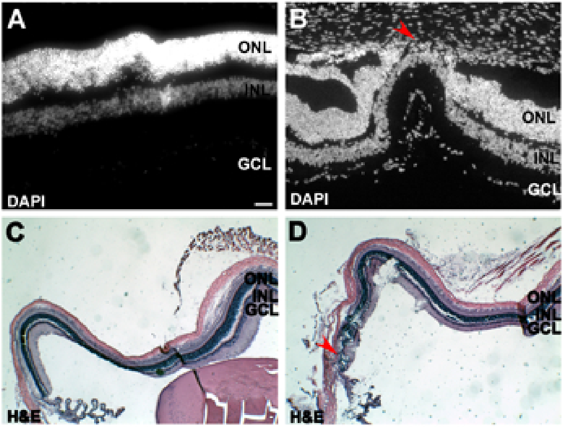

Touahri et al. Figure S1

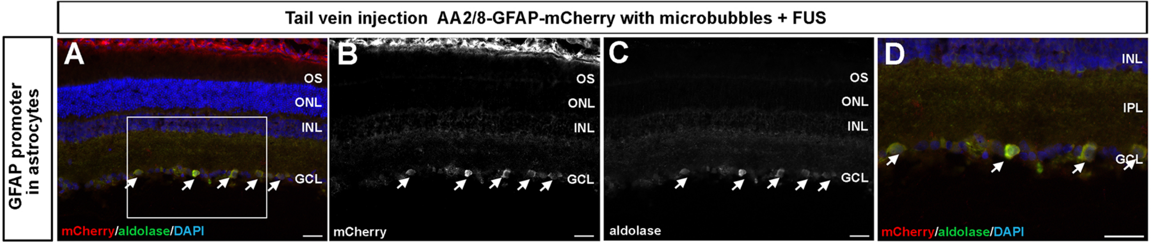

Touahri et al. Fig. S2

Supplemental Table 1. Pressure parameters in FUS experiments

Animal	Enhacement	Acoustic pressures (Mpa)
#1	No enhancement	0.7- 0.538- 0.675- 0.562
#2	No enhancement	0.650- 0.562- 0.737- 0.562
#3	Medium	0.712- 0.575- 0.7- 0.675
#4	Strong	0.9- 0.825- 0.795- 0.705
#5	Weak	0.660- 0.750- 0.765- 0.840
#6	No enhancement	0.772- 0.91- 0.822- 1.295

Supplemental Table 2. List of animals used in each experiment

PROCEDURE	Number of Animals	PURPOSE
**Systemic injection**		
AAV9-PHP-eB	3 mice	Retinal Transduction
rAAV2/8-GFAP-mCherry 2.5×109 GC/ml	3 mice	Peripheral Organ Transduction
PBS	3 mice	
**Retinal Explants**		
rAAV2/8-GFAP-mCherry 2.5×109 GC/ml	2 mice	Retinal Transduction - in vitro
**Intravitreal injection**		
rAAV2/8-GFAP-mCherry	3 rat eyes	Retinal Transduction - in vivo
PBS	3 rat eyes	
**MRIgFUS**		
Gadolinium/Evans Blue	6 rats	Permeabilization Assay In vivo
rAAV2/8-GFAP-mCherry 2.5×109 GC/ml	3 rats	Retinal Transduction - in vivo
rAAV2/8-GFAP-mCherry 1.25×108 GC/ml	3 rats	
